# Developing Entrustable Professional Activities for Dementia Care

**DOI:** 10.1002/alz70858_100681

**Published:** 2025-12-25

**Authors:** Huei‐Ling Huang, Pen‐Chen Kung, Ya Li Sung, Yen‐Ting Liao, Yen‐Fang Chou

**Affiliations:** ^1^ Chang Gung University of Science and Technology, Taoyuan, Taiwan; ^2^ New Taipei Municipal TuCheng Hospital, New Taipei City, Taiwan

## Abstract

**Background:**

Dementia has increasingly garnered attention and has become a global public health priority in recent years. To align school education with the needs of current practice settings, medical education has recently advocated for the use of “Entrustable Professional Activities” (EPAs) as a teaching tool to bridge the gap between academic knowledge and clinical practice. However, EPAs have been rarely applied in the field of dementia care. Therefore, we developed EPAs focused on dementia care, aiming to enhance the ability of professionals to provide care for individuals with dementia and their families.

**Method:**

A development team comprising dementia care experts was formed to create Entrustable Professional Activities (EPAs). The team reviewed relevant literature and discussed the essential tasks required for professional caregivers in dementia care. Using the nominal group technique, the team reached a consensus on task content. Clinical education experts with experience in developing or implementing EPAs were invited to evaluate the quality of three dementia care‐related EPAs. The evaluation was conducted using the Queen's EPA Quality Rubric (EQual), which employs a 5‐point scale to assess eight dimensions of the task descriptions.

**Result:**

A total of seven experts were invited to participate in the evaluation, including two nurse educators, one attending physician from the teaching department, one nursing supervisor, one director of nursing, and two faculty members. The average scores for the three EPAs were 4.18, 4.21, and 4.17, all exceeding the recommended threshold score of 4.07 on the rubric. This confirmed the quality of the three EPAs, which are as follows: (1) Assessing the need for community care resources for people with dementia, (2) Providing nursing health education on eating for people with dementia, and (3) Evaluating feeding issues for home‐based people with severe dementia.

**Conclusion:**

This study developed three dementia care‐related Entrustable Professional Activities (EPAs) through expert discussions and evaluations, demonstrating good quality. These EPAs can serve as a reference for enhancing the practical skills of professional caregivers and bridging the gap between academic education and clinical practice.

